# Assessment of the accuracy of patient-specific drilling guides for cervical (C5–C6) and lumbar (L4–L5) vertebrae in cats

**DOI:** 10.17221/73/2024-VETMED

**Published:** 2025-01-20

**Authors:** Rodrigo Casarin Costa, Gabriel Godoi de Moraes, Rafaela Gerbasi Ferreira, Renato Barroco Neto, Matheus Nobile, Thiago Andre Salvitti de Sa Rocha, Luciano Pereira de Barros, Bruno Watanabe Minto, Luis Gustavo Gosuen Goncalves Dias

**Affiliations:** ^1^Department of Veterinary Medicine and Veterinary Surgery, State University “Júlio de Mesquita Filho”/UNESP, Jaboticabal, SP, Brazil; ^2^Federal University of Jataí, UFJ, Jataí, GO, Brazil

**Keywords:** iatrogenic disease, neurosurgery, three-dimensional printing, vertebral body

## Abstract

Ten sets of vertebral biomodels and the corresponding drilling guides were created to evaluate the implantation accuracy in the cervical (C5–C6) and lumbar (L4–L5) vertebrae of cats. Smooth pins were implanted using the guides on the right side of five randomly assigned biomodel sets and on the left side of the remaining sets, with the contralateral side undergoing freehand implantation. Subsequently, a new tomographic study was conducted to measure the implantation angles. The pre-implantation angles were compared with the post-implantation angles between the techniques and among the sets. The guide-assisted implantation exhibited a lower dispersion compared to the freehand technique, with coefficients of variation of –1.95 and 48.9 in the cervical vertebrae and 1.98 and 9.39 in the lumbar vertebrae, respectively. However, no statistical difference was observed between the pre- and post-implantation angles, nor when comparing the vertebral segments (*P* > 0.05). Under the study conditions, the use of the guide failed to result in more accurate implantations in the C5–C6 and L4–L5 vertebral biomodels of cats.

Spinal trauma in feline patients has an incidence rate ranging from 3.4% to 13%, primarily caused by vehicular accidents and falls (Conroy et al. 2019; Keosengthong et al. 2019; Orgonikova et al. 2021a; Orgonikova et al. 2021b; Serra et al. 2021; Catalkaya et al. 2022). The treatment, with few exceptions, includes surgical reduction and stabilisation (Ozak and Inal 2016). One of the most used techniques is the insertion of screws into the vertebral body, combined with polymethylmethacrylate (Kim et al. 2014; Vallefuoco et al. 2014).

Iatrogenic injuries to the spinal cord, nerve roots, blood vessels, and intervertebral discs are among the primary intraoperative complications, along with early postoperative implant failure or loosening (Jeffery 2010; Orgonikova et al. 2021a; Orgonikova et al. 2021b). These complications are associated with the surgical planning and execution, specifically in identifying safe corridors in the vertebrae to ensure adequate bone stock and minimising the risk of injury to adjacent structures (Watine et al. 2006; Espadas et al. 2018; Kamishina et al. 2019).

Surgical planning is established by prior studies or individualised assessments through computed tomography (Rocha 2015; Martinelli et al. 2016; Hamilton-Bennett et al. 2017; Hsu et al. 2018; Burleson and DiPaola 2019). The execution of the planning is equally crucial; thus, tools, such as patient-specific drilling guides, have been developed to assist in this phase, thereby reducing the incidence of complications related to improper implant positioning (Rocha 2015; Martinelli et al. 2016; Hamilton-Bennett et al. 2017; Hsu et al. 2018; Burleson and DiPaola 2019).

Previous studies using prototyped drilling guides have shown greater accuracy in implant insertion, including a reduced surgical time and trauma in vertebral stabilisations in humans (Burleson and DiPaola 2019; Nanni et al. 2019) and dogs (Hamilton-Bennett et al. 2017; Fujioka et al. 2018; Kamishina et al. 2019; Toni et al. 2020).

Despite the recognised advantages of using prototyped drilling guides in other species, their application in feline vertebral stabilisation has not been fully explored. This study aimed to assess, through pre- and postoperative computed tomography, the accuracy of pin placement in vertebral bodies of biomodels, with or without the use of patient-specific drilling guides for cervical and lumbar spinal stabilisation in cats. The hypothesis was that the use of these guides could result in angles similar to those planned.

## MATERIAL AND METHODS

### Ethics

The study received approval by the Animal Use Ethics Committee of the Faculty of Agricultural and Veterinary Sciences (UNESP), under protocols 010197/19 and 010305/19.

### Model selection

**Figure 1:**
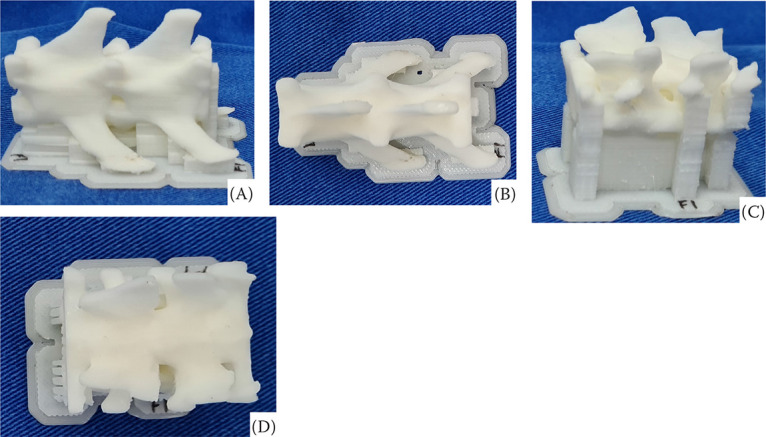
Photographic images of the biomodels of L4 and L5 and C5 and C6 vertebrae printed in an ABS filament from a CT scan of a domestic cat cadaver, used in the project execution Lateral view (A) and dorsal view (B) of L4 and L5 and lateral view (C) and ventral view (D) of C5 and C6

Ten cat cadavers (4.9 ± 0.6 kg) with no morphological alterations in the C5 and C6 cervical vertebrae and L4 and L5 lumbar vertebrae were selected. From these specimens, biomodels of the specified vertebrae were created using a 3D printer (model anycubic photon^®^ – Anycubic) (Figure 1).

### Division of the experimental groups

**Figure 2:**
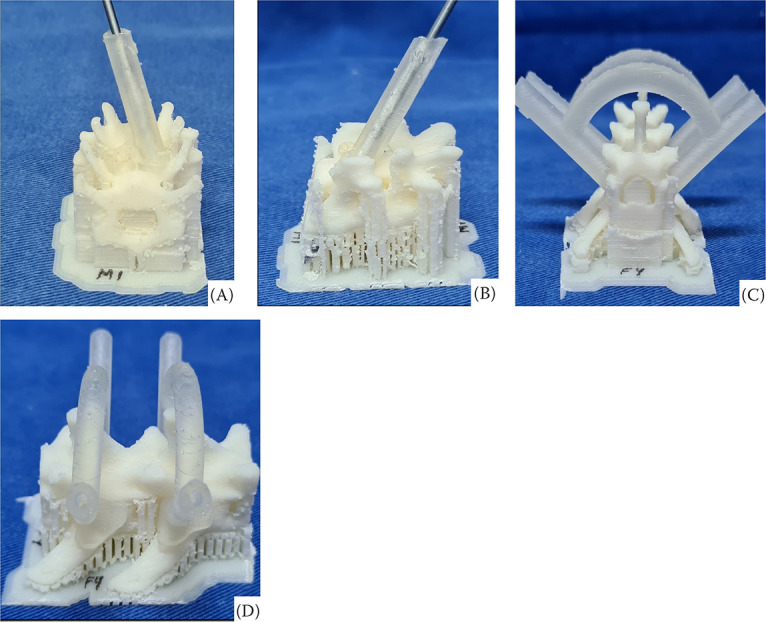
Photographic images of the biomodels and their respective guides Caudal view (A) and lateral view (B) of the cervical vertebrae. Cranial view (C) and lateral view (D) of the lumbar vertebrae

For the study, ten sets of C5–C6 and L4–L5 vertebrae were printed. These sets were divided into four groups: cervical freehand (CFH), cervical surgical guide (CSG), lumbar freehand (LFH), and lumbar surgical guide (LSG). The CFH and LFH groups utilised the right side of the vertebrae from five sets and the left side from the remaining five sets for implant placement without the aid of guides. Similarly, the CSG and LSG groups had five sets with implants placed on the right side and five on the left side, using specific drilling guides for each biomodel (Figure 2).

### Surgical planning and guide preparation

The selected cadavers underwent computed tomography, producing two-dimensional images in a DICOM (Digital Imaging and Communication in Medicine) format, which were then exported to the Slicer 3D^®^ software for three-dimensional reconstruction.

This software produced vertebral projections, and the images were transferred to RadiAnt^®^ and Blender^®^ software. In RadiAnt^®^, measurements of the entry and exit points, angulations, depth, and implant projections were conducted, along with the identification of each vertebra’s specific characteristics and irregularities for guide accommodation. In Blender^®^, the drilling guides for each vertebra were designed based on previously obtained anatomical measurements and specifications.

Subsequently, the file was exported to a Standard Tessellation Language format, where the virtual preparation of the guides was conducted, followed by their printing using a premium ABS filament via Repetier Host^®^ software and Melting 3D resin.

**Figure 3:**
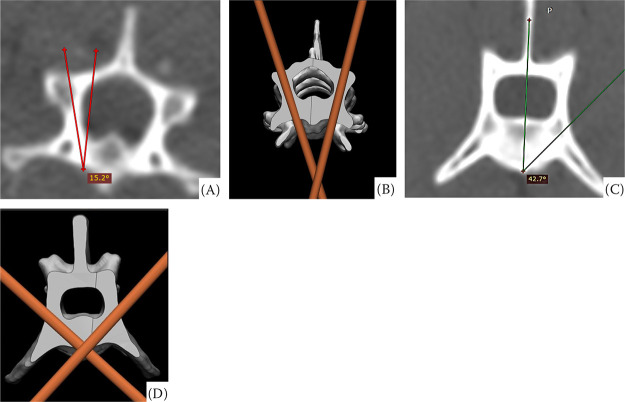
Images from the Radiant^®^ software (A and C) and Blander^®^ software (B and D) demonstrating the evaluation of safe corridors, the measurement of pre-implantation angles, and the simulation of the implant insertion in the cervical (A and B) and lumbar (C and D) vertebrae of a domestic cat during the surgical planning phase and the planning of the drilling guides

To measure the pre-implantation angles in the lumbar vertebrae, a straight line was drawn connecting the centre of the spinous process to the centre of the vertebral body, with a second line perpendicular to this line on the vertebral body, representing the safe drilling corridor. This configuration determined the desired angle for the implant insertion (Figure 3).

Using Radiant and Blander programs tools, measurements in the cervical vertebrae were done. A straight line was drawn connecting the base of the ventral tubercle, parallel to the centre of the vertebral canal, and a second line simulating the safe drilling corridor (Figure 3).

### Implantations

On the first day, the implantation technique was conducted using surgical guides, and on the following day, it was conducted freehand, with the side of the previously implanted vertebral sets covered with a sterile drape. The surgical implantation was performed by one person with neurosurgery experience and the sets preparation were conducted by others to reduce the interference of practical and visual experience in the results.

For the CSG and CFH groups, a ventral approach was used simulating ventral spine stabilisation and for the LSG and LFH groups, a dorsal approach was executed, simulating dorsal spine stabilisation.

In the CSG group, 2-mm diameter smooth titanium pins were allocated into the vertebral bodies of C5 and C6 using guides, oriented ventromedially to laterodorsally. The entry point for the implants was the ventral tubercle base, located in the cranial third of the vertebral body, on the right side of five specimens and on the left side of the remaining five. The guides fit into the ventral tubercle and the base of the transverse process of the vertebra, maintaining contact with the vertebral body.

In the LSG group, 2-mm diameter smooth titanium pins were inserted into the L4 and L5 vertebral bodies with the assistance of guides. The pins were placed in a laterodorsal to ventromedial direction, with the entry point at the junction between the pedicle and the base of the transverse process of the vertebra.

This procedure was performed on the right side of five specimens and on the left side of the remaining five. The guides were placed bilaterally to ensure higher stability, fitting into the pedicle and resting on the base of the transverse process and dorsally on the mamillary process.

In both the CFH and LFH, the implantation was executed without the aid of guides on the right side of five sets and on the left side of the remaining five sets, adhering to the pre-planned insertion point and angulation determined by computed tomography for each vertebra.

### Post-implantation assessment

Using a new computed tomography scan, the implantation angles were measured. The same reference lines from the planning phase were used; however, the safe corridor line was replaced by the central axis of the implant inserted into the vertebral body, thereby determining the post-implantation angle.

At this stage, the ∆ (delta) of the implantations was also calculated, representing the difference between the pre-implantation and post-implantation angles. This value reveals the variability between the planned and executed angles and can be either positive or negative. The closer the ∆ value is to 0, the more accurate the implantation compared to the planned procedure.

### Statistical analysis

All the statistical analyses were conducted by a data scientist using R software within the RStudio integrated development environment (v4.1.0; RStudio, Inc., Boston, USA).

The functions and packages used were specified in the ‘package::function’ format corresponding to the R programming language. The statistical significance was set at 5% for all the tests. The data were deemed to be non-parametric based on the Shapiro–Wilk and Levene tests. Consequently, the data were presented using the median, interquartile range, and coefficient of variation. Initially, the pre- vs post-intervention comparisons within the same treatment and group were conducted via a two-tailed paired Wilcoxon test. Subsequently, comparisons of the Δ between the treatments and between the groups were conducted using the two-tailed Mann–Whitney test.

## RESULTS

**Figure 4:**
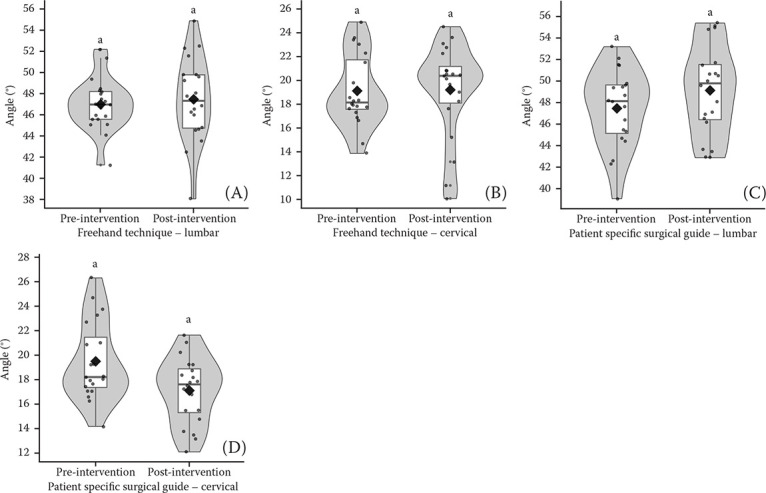
Box plot comparing pre- and post-intervention LFH (A), CFH (B), LSG (C), and CSG (D) The lowercase letter "a" shows that there is no statistical difference between the box plots CFH = cervical freehand; CSG = cervical surgical guide; LFH = lumbar freehand; LSG = lumbar surgical guide

The comparison of the pre- and post-intervention angles demonstrated no significant differences (*P *> 0.05) (Figure 4, Table 1). Although the coefficients of variation (CV) were low overall, the variability between the planned and executed angles was lower when using the guide compared to the freehand technique (Table 1).

**Table 1:** Median and interquartile range comparing the pre- and post-intervention angles for the guided and freehand treatments in the cervical and lumbar groups across 40 biomodels

Treatment	Group	Intervention	Median	IIQ	CV	*P*-value
Freehand	cervical (CFH)	pre	18.30	4.17	0.16	0.927 3
		post	20.55	3.10	0.21	
	lumbar (LFH)	pre	47.00	2.62	0.05	0.550 3
		post	47.35	5.02	0.08	
Guide	cervical (CSG)	pre	18.15	4.12	0.17	0.123 1
		post	17.55	3.60	0.16	
	lumbar (LSG)	pre	48.15	4.50	0.08	0.070 2
		post	49.80	5.12	0.08	

CV = coefficient of variation; IIQ = interquartile range

**Figure 5:**
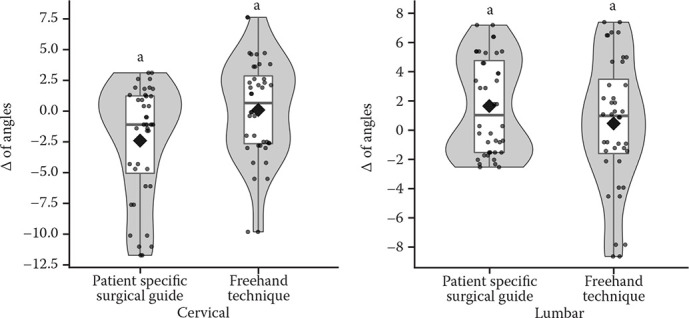
Box plot comparing the Δ between the guide and freehand groups for the cervical (A) and lumbar (B) segments The lowercase letter "a" shows that there is no statistical difference between the box plots

Regarding Δ, no statistically significant variation was identified between the CSG and CFH groups, nor between the LSG and LFH groups (*P *> 0.05) (Figure 5).

The use of guides, although not significantly more precise, exhibited less variability in the observations compared to the freehand technique, both in the cervical region (CSG vs CFH), with a CV of –1.95 and 48.9, respectively, and in the lumbar region (LSG vs LFH) with a CV of 1.98 and 9.39, respectively (Table 2).

**Table 2:** Median and interquartile range comparing the Δ between the guided and freehand treatments for the cervical and lumbar groups considering 40 biomodels

Group	Treatment	Median	IIQ	CV	*P*-value
Cervical	guide (CSG)	–1.10	6.27	–1.95	0.096 2
	freehand (CFH)	0.65	5.50	48.90	
Lumbar	guide (LSG)	1.05	6.27	1.98	0.560 8
	freehand (LFH)	1.00	5.07	9.39	

CV = coefficient of variation; IIQ = interquartile range

**Figure 6:**
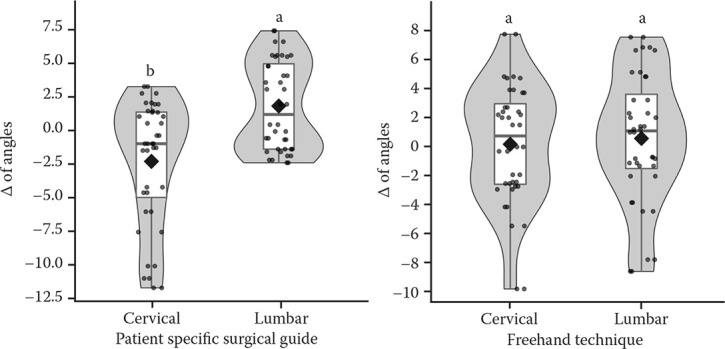
Box plot comparing the Δ between the cervical and lumbar segments in the surgical guide (A) and freehand (B) groups The lowercase letters "a" and "b" show whether there were statistical differences between the box plots

When comparing the accuracy of using the guide versus the freehand technique across segments by observing the variation in the respective deltas, it was noted that, with the guide, the lumbar perforations were more accurate (Δ closer to 0) than the cervical perforations (*P *= 0.02) (Figure 6A).

In the freehand technique, the cervical group and the lumbar group did not exhibit a significant difference (*P *> 0.05) (Figure 6B).

## DISCUSSION AND CONCLUSION

The use of patient-specific drilling guides has emerged as a prominent tool for implant placement in canine vertebrae (Hamilton-Bennett et al. 2017; Fujioka et al. 2018; Kamishina et al. 2019; Toni et al. 2020) and humans (Burleson and DiPaola 2019; Nanni et al. 2019), guaranteeing optimal bone stock and minimal trauma. This study critically evaluates the accuracy of these devices in the feline species. By comparing the reproducibility of the implantation planning and execution using the guide versus freehand techniques, with the hypothesis that the guides would lead to pre- and post-implantation angles more closely aligned than the freehand methods, no statistical relevance was noted in the study conditions.

No statistically significant difference in the accuracy was observed in either the cervical or lumbar segments compared to the freehand technique (*P *> 0.05) despite the reduced variability of observations and closer proximity to zero deltas when guides were used (Figure 6, Table 2). These findings align with Beer et al. (2020), who also found no superiority in the drilling accuracy in the lumbosacral vertebral segment of dogs between the same techniques (guide and freehand).

Opposing the findings of Bongers et al. (2022), it is believed that the surgeon’s experience influenced the lack of increased accuracy when using the guide compared to the freehand technique, as familiarity with the vertebral stabilisation techniques enhances the standardisation regarding the entry points and angulations.

Reinforcing this possibility, we also note the greater data dispersion when using the guide and freehand technique in the cervical spine, a vertebral segment with a lower incidence of traumatic injuries compared to the lumbar segment, indicating less familiarity among the surgeons between these segments (Saengthong et al. 2022).

The use of biomodels may have contributed to the low variability in the data by allowing the more accurate three-dimensional visualisation and positioning of perforations and vertebrae. This accuracy is achieved as biomodels do not replicate adjacent tissues that obscure anatomical references, thereby facilitating the angulation and fitting of guides, including the patient’s positioning on the table, as noted by Monteiro (2018).

The use of surgical guides, as demonstrated by previous studies, heightens the accuracy of the surgical execution through preoperative planning via computed tomography (Hamilton-Bennett et al. 2017; Fujioka et al. 2018; Burleson and DiPaola 2019; Kamishina et al. 2019; Nanni et al. 2019; Toni et al. 2020). These guides are strictly crafted to align with anatomical reference points, ensuring optimal accommodation, entry point accuracy, and desired angulation, thereby minimising the displacement and alteration during surgery

In the present study, a reduced dispersion of ∆ values relative to zero was noted when using the guide, with a cervical coefficient of variation (CV) of –1.95 compared to 48.9 for the freehand, and a lumbar CV of 1.98 and 9.39, respectively. However, under the study conditions, statistical significance was not attained. Perhaps with a greater number of observations, our results may achieve statistical values that confirm the guides’ accuracy as presented by other authors (Hamilton-Bennett et al. 2017; Fujioka et al. 2018; Burleson and DiPaola 2019; Nanni et al. 2019; Toni et al. 2020).

The insertion angles of the implants obtained during the pre-implantation planning in the lumbar vertebrae showed a median of 47°, aligning with the literature for canines, which indicates angles ranging from 45° to 60° (Watine et al. 2006). However, this does not align with the limited studies in felines that recommend angles of 90° (Vallefuoco et al. 2014). A 90° angle guarantees a greater bone stock compared to angles between 45° and 60°, but further studies are warranted to verify the actual biomechanical resistance gain between the two angles concerning the bone stock.

Conversely, a median angle of 18° was identified in the cervical vertebrae, diverging from the limited data found in the literature for the feline species, which ranges from 47° to 70° (Espadas et al. 2018). The discrepancy between the studies is attributed to the selected entry point on the vertebral body: in the present study, the base of the ventral tubercle of the vertebrae was used as the entry point, whereas in the limited published works, the centre of the vertebral body was employed as the reference for the vertebrae in question (C5 and C6) (Espadas et al. 2018).

In the study design, a 2-mm diameter smooth pin was selected owing to its superior resistance compared to the biomodel material, thereby minimising the interference of material deformation on the obtained angulation during drilling. The objective was to evaluate the execution of the planning. As indicated by a previous study, 2-mm pins are overestimated for feline cervical vertebrae, with an 87% and 75% risk of injury in C5 vertebrae and C6 vertebrae. respectively (Espadas et al. 2018). Consequently, the possibility of spinal canal invasion among the used techniques was not evaluated.

The data, regarding the variation in safe corridors and the diameter of implants to be used, underscore the requirement for further studies on the implant-anatomy relationship in the feline vertebrae, including the importance of tomographic planning for each case.

The authors identify numerous limitations of the study that could potentially alter the results, such as the comparison between the implantations performed by surgeons with differing levels of neurosurgical experience, the evaluation of the bone stock acquired through drilling as a clinical evaluation of a pedicle breach using an adequate implant diameter, the use of a single experimental model for biomodels to minimise any individual anatomical variation, and the application of the same methodology on cadavers to determine the influence of adjacent tissues at the operative site on the techniques studied.

Under the study conditions, the use of patient-specific surgical guides developed from 3D printing, based on tomographic studies of the cervical (C5 and C6) and lumbar (L4–L5) vertebrae in domestic cats, did not guarantee greater accuracy compared to the freehand technique.
